# Effects of Laser Application on Alveolar Bone Mesenchymal Stem Cells and Osteoblasts: An In Vitro Study

**DOI:** 10.3390/diagnostics12102358

**Published:** 2022-09-29

**Authors:** Luminița Lazăr, Doina Ramona Manu, Timea Dako, Maria-Alexandra Mârțu, Mircea Suciu, Alina Ormenișan, Mariana Păcurar, Ana-Petra Lazăr

**Affiliations:** 1 Department of Periodontology, George Emil Palade University of Medicine, Pharmacy, Science, and Technology of Târgu Mures, 38 Ghe. Marinescu Street, 540139 Târgu Mures, Romania; 2 Center for Advanced Medical and Pharmaceutical Research, George Emil Palade University of Medicine, Pharmacy, Science, and Technology of Targu Mures, 540142 Târgu Mures, Romania; 3 Department of Odontology and Oral Pathology, George Emil Palade University of Medicine, Pharmacy, Science, and Technology of Târgu Mures, 38 Ghe. Marinescu Street, 540139 Târgu Mures, Romania; 4 Department of Periodontology, Grigore T. Popa University of Medicine and Pharmacy Iasi, Universitatii Street 16, 700115 Iasi, Romania; 5 Department of Oral Rehabilitation and Occlusology, George Emil Palade University of Medicine, Pharmacy, Science, and Technology of Târgu Mures, 38 Ghe. Marinescu Street, 540139 Târgu Mures, Romania; 6 Department of Oral and Maxillofacial Surgery, George Emil Palade University of Medicine, Pharmacy, Science, and Technology of Târgu Mures, 38 Ghe. Marinescu Street, 540139 Târgu Mures, Romania; 7 Department of Orthodontics, George Emil Palade University of Medicine, Pharmacy, Science, and Technology of Târgu Mures, 38 Ghe. Marinescu Street, 540139 Târgu Mures, Romania; 8 Institution Organizing University Doctoral Studies (I. O. S. U. D.), George Emil Palade University of Medicine, Pharmacy, Sciences and Technology of Târgu Mureş, 38 Ghe. Marinescu Street, 540139 Târgu Mures, Romania

**Keywords:** laser application, mesenchymal stem cells, osteoblasts, alveolar bone, cellular activity

## Abstract

Mesenchymal stem cells isolated from the bone marrow have a great differentiation potential, being able to produce many cell lines, including osteoblasts. Osteoblasts have an important role in bone remodeling by actively participating in the maturation and mineralization of the extracellular matrix. The aim of this study was to determine the effect of laser application on the viability and proliferation of osteoblasts. Methods: Alveolar bone was harvested from 8 patients and placed into a culture medium to induce proliferation of mesenchymal stem cells. These were differentiated into osteoblasts in special conditions. The cells from each patient were split into two groups, one was treated using a 980 nm laser (1W output power, pulsed mode, 20 s, 50 mm distance) (laser “+”) and the other one did not receive laser stimulation (laser “-”). Results: Using the confocal microscope, we determined that the cells from the laser “+” group were more active when compared to the laser “-” group. The number of cells in the laser “+” group was significantly greater compared to the laser “-” group as the ImageJ-NIH software showed (*p* = 0.0072). Conclusions: Laser application increases the proliferation rate of osteoblasts and intensifies their cellular activity.

## 1. Introduction

Human mesenchymal stem cells (HMSc) were first described by Friedenstein et al. as cells that have a high multiplication potential [1]. The same team studied stem cells for the next 20 years and demonstrated their usefulness in the restoration and regeneration of cartilaginous, adipose, but especially bone tissues. Currently, stem cell research is an extremely active field with a dynamic evolution [2,3,4]. Many clinical studies have been conducted regarding the use of stem cells in the treatment of autoimmune diseases, tissue damage or regenerative therapies. The anti-inflammatory and immunosuppressive capacity of mesenchymal stem cells improve clinical results of numerous therapies [5,6]. Thus, the use of bone-derived mesenchymal stem cells (BDMSCs) can have multiple beneficial effects [7,8].

Mesenchymal stem cells (MSCs) can originate from various organs such as the lungs, intestines, liver, and bone marrow. MSCs isolated from the bone marrow have a great differentiation potential, being able to produce many cell lines such as chondroblasts, adipocytes, fibroblasts, cardiomyocytes, keratinocytes, hepatocytes, neural cells, or osteoblasts [9,10,11,12]. Differentiation towards one lineage or another occurs according to specific culture conditions that mimic the conditions these cells experience in vivo. Obtaining MSCs from bone marrow is a simple procedure that is followed by cell proliferation in vitro. Once cultured, these cells require specific stimulation to avoid unwanted effects such as premature cellular aging or inactivity [13,14,15]. Cellular metabolism is normally influenced by interactions with other tissues and cells. When they are isolated and cultured, the nutritional requirements change and may vary between cells [16,17,18].

Researchers and clinicians are increasingly motivated to use mesenchymal stem cells of dental origin (SHED) in tissue regeneration therapies [19,20,21]. They are a sub-group of mesenchymal stem cells and represent a population of postnatal cells with the ability to differentiate into various cell types. Such cells can be obtained at the time of interventions from the bone level, without requiring other invasive procedures for harvesting. Mesenchymal stem cells of dental origin have been shown to have significant differentiation potential, an increased proliferation rate, and also the ability to mineralize the extracellular matrix [22,23]. Moreover, dental-derived MSCs (DMSCs) show the same features as bone marrow-derived MSCs (BM-MSCs) and exert immunomodulatory and anti-inflammatory effects through tumor necrosis factor-α, platelet-derived growth factor, interleukin-1 and IL-6, initiating DMSCs activation and recruitment. 

Other cell lineages such as embryonal stem cells (ESC) and induced pluripotent stem cells (iPSC) have been actually used only in in vitro and in vivo animal studies. iPSC research promises great potential for dental tissue regeneration thanks to its similar characteristics to embryonal stem cells, and lack of ethical issues. Although many advantages of iPSC use are foreseen, there are still several challenges, which have to be overcomed before clinical application, such as teratoma formation and malignant transformation [24].

Tetè et al. also showed that ESCs and MSCs have a lower risk of carcinogenesis and hyperproliferation than iPSCs and that osteogenic differentiation of iPSCs with bone grafts has had limited results. In their work, four iPSCs clones were successfully obtained and further differentiated into osteoblast cells following the protocol which uses osteogenic differentiation medium supplemented with ascorbic acid, β-glycerophosphate and 10 nm dexamethasone [25,26].

In fact, it has been shown that iPSCs could be differentiated into MSCs (iPSC-MSCs) with benefits over direct differentiation of iPSCs into osteoblasts. iPSC-MSCs have the same osteogenic potential as MSCs derived from other sources, such as bone marrow or dental pulp. iPSC-MSCs have a reduced risk of tumor formation [27].

Therefore, an ideal technique to regenerate damaged bone tissue must be found. The most promising perspectives are stem cell-based techniques with mandatory use of the most convenient combination of cells, signaling molecules and bone regeneration scaffolds.

Osteoblasts are cuboidal cells of mesenchymal origin, rich in cytoplasmic organelles, specialized in the formation and secretion of extracellular matrix (ECM) and which then actively participate in its maturation and mineralization [28]. These cells have a relatively short lifespan of about 3 months in human bones. Due to the fact that bone tissue undergoes a continuous process of remodeling, osteoblasts are always present at this level. Once an osteoblast ends its activity, there are three possible options:

− They become osteocytes embedded in mineralized ECM and lose most of their cytoplasmic organelles. Osteocytes have low metabolic activity, can be present throughout the patient’s entire life, and represent approximately 90–95% of bone cells in the adult [29].
− They die by apoptosis.
− They turn into BLC (bone lining cells), a line of post-mitotic osteoblasts, with a flat appearance that are found on the surface of the bone. These cells have an important role in regulating bone remodeling processes [30].

Consequently, new osteoblasts will arise from the mesenchymal stem cells present in the bone marrow, and thus the process resumes. The mesenchymal stem cell population actively proliferates in the initial stages of osteogenesis. As they begin to differentiate into osteoblasts, the proliferation rate decreases, and osteogenic markers begin to appear. Alkaline phosphatase is produced by young osteoblasts (matrix maturation phase), and osteocalcin is secreted by mature osteoblasts (matrix mineralization phase) (Figure 2) [31]. 

In vitro differentiation of osteoblasts goes through three distinct phases: proliferation, maturation of the extracellular matrix, and its mineralization. As the differentiation process takes place, the level of alkaline phosphatase increases, which leads to the conversion of organic phosphate to inorganic phosphate. The result of this process is the formation of hydroxyapatite, a mineral that is deposited at the level of the extracellular matrix [32].

During orthodontic treatment, remodeling phenomena occur in the periodontal tissues, most importantly in the surrounding bone tissue. Bone tissue has a remarkable capacity for self-repair and regeneration. In the process of bone regeneration, the interaction between different cells, such as inflammatory cells, osteoclasts, and osteogenic cells, is necessary. Pre-osteoblastic cells and osteoblasts are the main cells responsible for the continuous development and creation of new functional tissue in the bone matrix [33].

Since its first applications in dentistry, the laser has been used for various procedures, such as hard and soft tissue surgery, biostimulation, disinfection of periodontal pockets, calculus removal, treatment of peri-implantitis, treatment of herpes lesions and aphtae. Among these, biostimulation has the most potentially intriguing effects by applying a low-energy laser beam to the tissues, for the purpose of achieving a biological effect. These effects can vary between accelerated wound healing, accelerated orthodontic movement and pain reduction [34,35,36,37]. 

In order to obtain cell cultures, we collected bone material from adult patients who frequently require periodontal, implantary or orthodontic treatment involving changes in the bone tissue. Primary cells provide a more complex response and faster feedback to environmental stimuli in vitro. Therefore, the fact that we used human bone cells and not commercial cells gives a greater importance to our results.

Since the activity of osteoblasts is paramount to periodontal regeneration but also bone healing, the purpose of this study was to assess whether laser stimulation has an effect on the viability and proliferation of osteoblasts originating from a dental extraction socket.

## 2. Materials and Methods

### 2.1. Patient Selection

For the purpose of this study, 10 patients were selected, between November 2021 and June 2022, who met the following inclusion criteria: age between 18 and 55 years, with carious lesions in premolars and molars, which had therapeutic indication for extraction, and post-extraction management required a regularization of the alveolar bone ridge or removal of a fractured bone fragment. The exclusion criteria were the detection of any signs of active periodontal disease and the presence of systemic diseases with impact on the periodontal tissues (diabetes, immunological diseases, acute articular rheumatism, tuberculosis), use of antibiotics, non-steroidal anti-inflammatory drugs or other medication which can interfere with bone metabolism in the last 3 months, pregnant or lactating women.

### 2.2. Bone Tissue Harvest

After anesthesia and tooth extraction, maxillary/mandibular bone tissue was harvested with bone nippers, by either removing a bone fragment fractured during the extraction or by regularizing the edentulous ridge. Bone tissue was collected in 15 mL Falcon tubes (Nerbe Plus, cat. no. 02-502-3001), in sterile DMEM culture medium (Sigma, StableCell™ Dulbecco’s Modified Eagle’s Medium high glucose, cat. no. D0819) supplemented with 1% antibiotics (Penicillin-Streptomycin, Sigma cat.no. P4333).

### 2.3. Bone Explant Cultivation

Within 3 h after harvesting, the bone explant was cultured after cleaning the bone tissue fragments with DPBS saline phosphate buffer (Sigma cat. no. D8662) to remove red blood cells and fragments from other types of tissues. The fragments were introduced into the culture medium with 10% FBS (Fetal Bovine Serum, Sigma Aldrich F7524, Burlington, MA, USA) and 1% antibiotics (100 U/mL penicillin and 100µg/mL streptomycin, Sigma cat.no. P4333) and centrifuged at 4000 rpm, for 10 min at room temperature to mobilize cells from the stromal tissue. The bone fragments and the medium were transferred to a T-25 cell culture flask (Eppendorf^®^ Cell Culture Flask T-25, cat.no. EP0030710126), cultivated as explants at 37 °C, 5% CO_2_, in 100% humid atmosphere. After 7 days, the medium is replaced with an equal volume of fresh medium supplemented with 10% FBS and 1% antibiotics, without dislocating the explants. The migration of mesenchymal stem cells from the tissue was observed under a microscope after 7–10 days. The cultures must reach the confluence required for sub cultivation within 4–6 weeks after cultivation. Adhered cells, with 80–90% confluence, in the log phase of proliferation were detached with trypsin/EDTA solution containing collagenase (StemCell Technologies Accutase, cat. no. 07920). The number and viability of the cells was checked under the microscope, subculturing was done at a density of 5 × 104 cells/cm^2^ and after the cells reached confluence, they were cryopreserved at a density of 1–2 × 106 cells/mL cryopreservation medium with 10% cryoprotectant DMSO (Dimethyl sulfoxide, Sigma Aldrich cat. no. 276855).

### 2.4. Differentiation of Mesenchymal Stem Cells to Osteoblasts under Laser Application

Thawing of the cells was done briefly, at 37 °C, for 1–2 min, for osteoblast differentiation, in the specific culture medium, with or without the action of laser. Mesenchymal stem cells isolated by the culture of bone tissue explants from the jaw bone were cultured in osteoblast differentiation medium containing: Alpha Minimal Essential Medium (Sigma Aldrich M4655) with 10% Fetal Bovine Serum (FBS, Sigma Aldrich F7524), 50 μg/mL Amphotericin B (Sigma Aldrich A2942), 25 μg/mL Gentamicin (Sigma Aldrich G1397), 50 μg/mL L-ascorbic acid (Sigma A4403), 10 mM of β-Glycerophosphate disodium salt hydrate (Sigma Aldrich G9422) and 10^−8^ M Dexamethasone (Sigma Aldrich D4902) at 37 °C, in an atmosphere with 100% humidity and 5% CO_2_. Cultivation for osteoblast differentiation was done on separate coverslips in culture chambers, for analysis in a confocal equipment (Cell Imaging Coverglass Eppendorf cat.no. 0030742028), at an initial density of 5 × 10^4^ cells/chamber. 

The cells obtained from each patient were cultured in two ways: the laser group “+” were cultures to which laser application was applied and the laser group “-”, cultures to which laser application was not applied. The laser exposure was performed with a dental laser (Prime, Litemedics, Lambda SpA, Milano, Italy), with a power of 1 Watt, in a pulsed system and operating wavelength 980 nm, set for the therapy, bio-stimulation working mode. The 320 μm optical fiber was held at a distance of 50 mm from the culture chamber, perpendicular to its surface, for 20 s.

For each patient in the laser “+” group, the culture chamber was exposed to laser irradiation at time T0 (24 h after osteoblast cultivation) and time T1 (3 days after T0).

### 2.5. Differentiation of Mesenchymal Stem Cells into the Osteoblast Phenotype

For osteoblast differentiation analysis, adherent cells, proliferated at 80–90% confluence, were fixed with a solution containing 4% paraformaldehyde and saponin (Becton Dickinson Cat. No. 554714) for 15 min at room temperature. The fixing solution was removed, and the cells were washed with buffer containing saponin (Becton Dickinson Cat. No. 554723), to permeabilize the cellular lipid bilayer, favoring the access of antibodies to antigens. Blocking of the non-specific sites was done with DPBS containing bovine serum albumin (BSA, Capricorn Scientific, cat. no. BSA-PF-1U) in a concentration of 0.3% and normal goat serum (NGS, ABCAM cat. no. ab7481) in a dilution of 1:50 for 10 min, at room temperature. Cells were stained with primary antibodies: Osteocalcin Monoclonal Antibody (Invitrogen OC4–30, Waltham, MA, USA) and Alkaline Phosphatase (ALPL) Recombinant Rabbit Monoclonal Antibody (Invitrogen 7H11L3, Waltham, MA, USA) overnight, at 4 °C. Alexa Fluor 488 Goat Anti-Rabbit IgG (Invitrogen H+L, Waltham, MA, USA) and Alexa Fluor 594 Goat Anti-Mouse IgG (Invitrogen H+L, Waltham, MA, USA) were used as secondary antibodies, to observe osteogenic differentiation. For autofluorescence negative controls, cells were incubated with buffer only, and for non-specific labeling controls, cells were exposed to secondary antibodies only. The nuclei were counterstained with 4′,6-diamidino-2-phenylindole (DAPI, ThermoFisher Scientific Cat. No. D1306). Images were acquired in TCS Leica SP8 confocal equipment, with the LASX application. The confocal equipment settings are shown in Table 1. The excitation source of the fluorochromes used is Argon laser for Alexa Fluor 488 and DPSS 561 for Alexa Fluor 594. For DAPI (4′,6-diamidino-2-phenylindole) the excitation source is Diode 405. The emitted signals came from a 1.16 × 1.16 µm surface. The matrix size is 1024 × 1024 pixels. Acquisitions were made with a dry N PLAN 10×/NA = 0.25 objective. The emission wavelengths pass through a mirror slit of tunable width, which selects the emission band. Photons emitted by Alexa Fluor 488 and DAPI molecules were collected on a photomultiplier (PMT), and photons emitted by Alexa Fluor 594 were collected on an internal hybrid detector (HyD). Once established, the sensitivity settings of the confocal system were kept unchanged during all acquisitions.

### 2.6. Analysis of the Viability and Proliferation of Osteoblasts under the Effect of Laser Application

For cell viability experiments without and with laser exposure, the cells were cultured under the same conditions. Viability analysis has been performed using the viability/cytotoxicity kit with Calcein AM and Homodimer Etidium III (Biotium Viability/Cytotoxicity Assay Kit for Animal Live & Dead Cells, cat. no.30002-T). The optimal concentration of Calcein viability reagent was established, because it may vary depending on the cell type (the level of esterase activity varies in the different cell types). Through optimization, the concentration of EthD that stains the nuclei of dead cells in deep red was selected, without significantly staining the nucleic acids in the cytoplasm of living cells. Mesenchymal stem cells were cultured under the same conditions to highlight alkaline phosphatase and osteocalcin, as well as viability, in osteoblast differentiation medium and at the same initial density of 5 × 104 cells/culture chamber.

Cells were incubated with Calcein AM/EthD-III solution for 30 min at room temperature and in the dark. After staining, the Calcein AM/EthD-III solution is replaced with DPBS and the acquisition in the confocal equipment is done immediately, according to the settings in Table 2. 

In the Leica TCS SP8, the excitation source of the fluorochromes used, Calcein and Ethidium III Homodimer, is Argon and DPSS 561 laser, respectively, and the excitation wavelengths were 496 and 561 nm. Acquisitions were made with a dry N PLAN 10×/NA = 0.25 objective. The emitted signals come from an area of 2350 μm × 2350 μm, being acquired from 3 × 3 fields of view (FOV), with the Tile Scan function from the LAS X application of the confocal equipment. The evaluation of cell viability and proliferation was constantly done from the same region of the culture chambers, by keeping the coordinates of Position 1, around which the 3 × 3 acquisition FOV was set, constant. The matrix size is 1024 × 1024 pixels. Photons emitted by Calcein molecules were collected on HyD, and those emitted by Propidium Iodide were collected on PMT.

### 2.7. Quantification of Cell Proliferation under the Effect of Laser

Data from images acquired with the confocal equipment were analyzed using the Analyze Particles command in the ImageJ-NIH software (https://imagej.nih.gov/ij, accessed on 28 January 2020).

To perform cell counting, the images were first converted to grayscale. To distinguish cells from the background, a threshold is set to suppress background pixels, eliminating intensities above the threshold. The result is a binary image where all the object pixels are black, while the background is white. To identify which pixels represent cells in images, any cluster of pixels that is too small to be a cell must be ignored. For this purpose, a representative circle can be drawn and measured for the smallest cell in the image using the ROI Manager. The Watershed command helps identify adjacent or overlapping groups of cells. Cell counting was finally performed using the Analyze Particles function. From the acquisition area of 2350 μm × 2350 μm, the groups of pixels having at least the area of the smallest object (smallest fluorescent cell) in the image were counted.

### 2.8. Statistical Analysis

All data was collected in Microsoft Excel work sheets (Microsoft Corporation, Washington, DC, USA, 2018). The statistical analysis was carried out in GraphPad Prism version 8.0.0 for Windows (GraphPad Software, San Diego, CA, USA). For each group of data, descriptive statistics such as mean, standard deviation, median, minimum, and maximum value, were assessed. Data normality was determined using the Kolmogorov–Smirnov test. The difference regarding the cell numbers between the laser “+” and laser “-” groups were determined using the t Student test. The chosen significance level was set at 0.05.

## 3. Results

The patients included in this study had certain age-related characteristics, being represented by 5 women and 3 men (Table 3).

**Figure 1:**
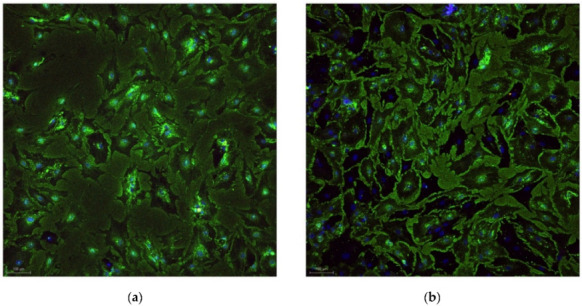
Confocal marker identification of osteoblasts stained for alkaline phosphatase and osteocalcin.: (**a**) laser “+” and (**b**) laser “-”.

**Figure 2:**
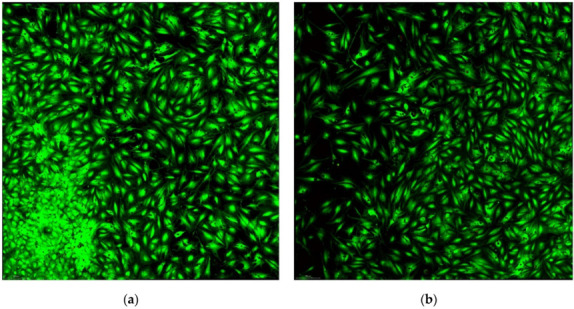
Osteoblast cell viability: (**a**) laser “+” and (**b**) laser “-”.

The sets of images acquired in the confocal equipment, to highlight osteoblast differentiation markers, alkaline phosphatase, and osteocalcin, for each of the 8 patients (laser “+” and laser “-”) are presented in Figure 1 and Figure 2.

**Figure 3:**
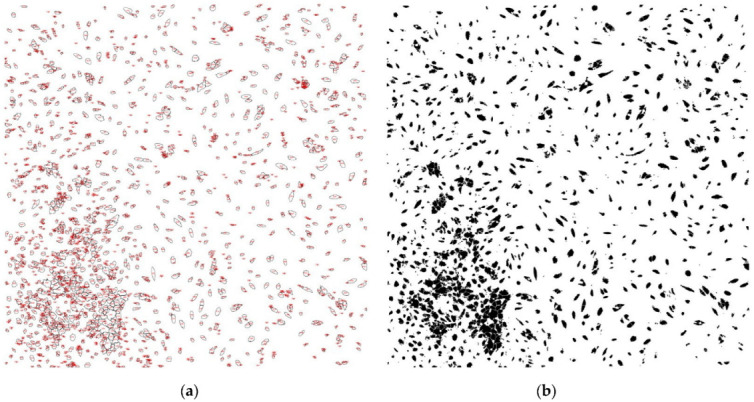
Quantification of cell proliferation (laser “+”): (**a**) cell counting (**b**) binary.

**Figure 4:**
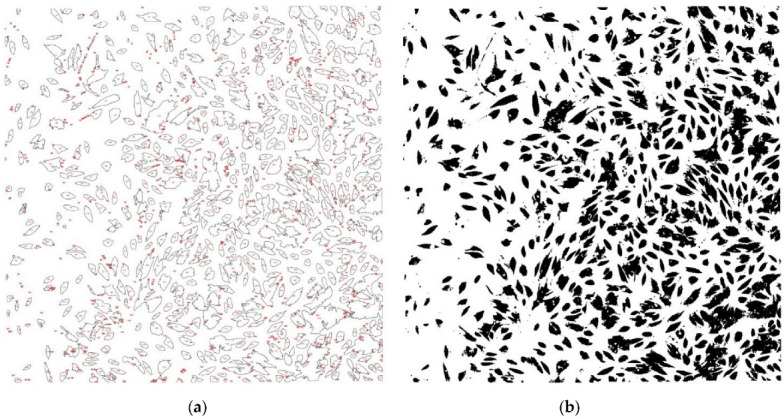
Quantification of cell proliferation: (laser “-”) (**a**) cell counting (**b**) binary.

When counting the cells, performed using the Analyze Particles function, in the acquisition area of 2350 μm × 2350 μm, we obtained the values contained in Table 4 and illustrated in Figure 3 and Figure 4.

## 4. Discussion

For this study we chose adult patients whose therapeutic indication would be extraction and that would require post-extraction regularization of the alveolar bone ridge or removal of a fractured bone fragment. In this way, the harvesting of bone tissue was done for the benefit of the patient, favoring post-extraction healing. In order to have a healthy bone fragment available, we excluded patients who had active periodontal disease or who had systemic conditions or medication that could impact the periodontal tissues.

Although the harvesting and culturing protocol was similar for each patient, isolated mesenchymal stem cells from one patient did not survive to differentiate into osteoblasts, so we excluded this patient from the laser irradiation protocol.

In our study the parameters for laser exposure were chosen to be comparable to those most commonly used in current practice. We used a LASER device with a power of 1 Watt, in pulsating system and operating wavelength 980 nm, setting it at the working mode therapy and bio-stimulation. The exposure time was 20 s; initially, the 320 μm optical fiber was held at a distance of 20 mm from the culture chamber, perpendicular to its surface. Because we noticed that most of the cells died, when assessing the cell viability for the first patient, we excluded this patient from the study and for the following patients we modified the working protocol, applying the irradiation from a distance of 50 mm, without removing cells from the culture medium. We explained this by the fact that in vitro, the cells were directly exposed to irradiation, without having any protective shield as they do in vivo.

When quantifying cell proliferation, we identified a greater number of cells in the cultures from the laser group “+” compared to the laser group “-” for all patients, with statistically significant difference (*p* = 0.0072). Cell proliferation was different from one patient to another, even though the working protocol was identical. This was influenced by the patient’s age; younger patients aged between 20 and 35 showed increased cell proliferation in both the “+” laser group and the “-” laser group, compared to patients aged between 35 and 50 years. The lowest values were observed in a 49-year-old patient, which can also be correlated with the bone structural changes generated by menopause.

Regarding the total area occupied by cells in the culture chambers, in 75% of the patients it was higher in the “+” laser group than in the “-” laser group. The average size of the cells was smaller in most patients (5 out of 8) for the “+” laser group than those in the “-” laser group. This is explained by the fact that in these patients there was a greater number of cells, which makes them crowded and, therefore, smaller in size. The area on the culture chambers occupied by adherent and proliferating cells, expressed as a percentage, was higher in the “+” laser group than in the “-” laser group for 6 out of 8 patients. For patients 1 and 2 for whom the area percentage of the coverslip covered by cells was lower, i.e., equal between the laser groups “+” and the laser group “-”, we identified groups of joined or overlapped cells in the analysis of the binary image, using the Watershed command.

Osteogenesis is the result of biochemical functions triggered by the induction of mesenchymal stem cells at sites of bone remodeling, which are activated by secretory transforming growth factor beta 1 (TGFβ). The cells differentiate into osteoblasts with the capacity for proliferation, further differentiation, specialized cell marker expression, collagen secretion and matrix mineralization [33].

Infante et al. stated that the mesenchymal stem cell population in the initial stages of osteogenesis is highly proliferative, but as they differentiate into osteoblasts, the proliferation rate decreases, and osteogenic markers begin to appear. These markers are alkaline phosphatase (secreted by young osteoblasts—matrix maturation phase) and osteocalcin (secreted by mature osteoblasts—matrix mineralization phase) [31]. In our study, when analyzing the cells at 7 days in the confocal equipment, we observed according to fluorochrome AlexaFluor 488 emission, that alkaline phosphatase antigen is expressed in a much higher amount than osteocalcin. Thus, the osteoblasts studied by us, being in the early phase of differentiation, produce more alkaline phosphatase than osteocalcin.

Low-level laser application (LLLT) or photo biomodulation (PBM) uses a low-power light source, which favors reparative phenomena in tissues, decreases inflammation and has an analgesic effect [38]. PBM thus activates redox biochemical reactions, which modulate the oxidative states of atoms. Secondarily, changes also occur in the cell’s metabolism, which affect the cell’s behavior as well [39,40]. Although a number of in vivo and in vitro studies have demonstrated the beneficial effects of using LLLT, its widespread use is controversial, due to the lack of standardization of the parameters (exposure time, frequency of exposures, wavelength) that influence the dose [41,42,43].

A study by Hiromi et al. aimed to evaluate the effects of LLLT on the proliferation and osteogenic differentiation of osteoblast-like cells isolated from the skull of 3–5-day-old Wistar rats. Cells were irradiated with 2.2, 3.3 and 4.3 J/cm^2^. After deletion, cell proliferation was assessed by flow cytometry and CCK-8. Calcification was assessed by measuring alizarin red S staining areas after 7, 14 and 21 days of culture in osteo inductive medium. Gene expression in non-irradiated and laser-irradiated cells was assessed by qPCR at 3, 6, and 12 h, as well as 1, 3, 7, and 14 days after irradiation. Microarray analysis was performed to comprehensively assess the gene expression of non-irradiated and 3.3 J/cm^2^ irradiated cells 6 h after irradiation. No significant increase in cell surface temperature was induced by irradiation. Irradiation did not affect the proliferation of osteoblastic cells. Calcification of osteoblast-like cells increased significantly 7 days after laser irradiation at 3.3 J/cm^2^. Bglap expression was significantly increased in cells irradiated at 3.3 J/cm^2^ at 6 h after irradiation. Microarray analysis showed that irradiation at 3.3 J/cm^2^ caused up-regulation of inflammation-related genes and down-regulation of Wisp2. Gene set enrichment analysis also clarified the enrichment of inflammation and Notch signaling related gene sets. In conclusion, low-level laser irradiation at 3.3 J/cm^2^ enhanced the calcification of primary osteoblast-like cells through enhanced Bglap expression and an enriched Notch signaling pathway [44].

A group of Brazilian researchers conducted a literature review on the impact of photo biomodulation on osteoblast cultures. Searches were made in several databases—PubMed/MEDLINE (Medical Literature Analysis and Retrieval System Online), SCOPUS, and SPIE digital library—for articles published in English, in the last 20 years, regarding the effects of LLLT on osteoblastic cells. 1439 studies were found, out of which, after the abstract analysis, 1409 were excluded and 30 were fully reviewed. Finally, after a critical analysis, 22 studies remained; despite the variety of the experimental model (the cell lines studied were human primary, rat primary, saos-2, Osteo-1, MC3T3, MG63, and OFCOL II), the same methods were used (alkaline phosphatase, MTT and cell count) to analyze the impact of LLLT on cells. This review suggests that osteoblastic cells are susceptible to photo-bio-stimulation, and the small differences between the irradiation parameters used by different authors have no influence on cell proliferation, while the use of high levels of irradiation have demonstrated harmful effects on proliferation [45].

Li et al. conducted a study to evaluate the effects of low intensity laser irradiation on cell proliferation in vitro. The cells were examined by flow cytometry, and the results showed that laser irradiation induced cell proliferation and transformation into osteoblasts, compared with the control group [46].

Chang et al. comparatively investigated the effects of laser irradiation, with a wavelength of 630 nm and 810 nm, on pre-osteoblastic cell cultures. Flow cytometric analysis, alkaline phosphatase (ALP) staining, ALP activity, Alizarin Red S staining, and quantitative analysis by real-time polymerase chain reaction (qRT-PCR) were performed to assess treatment response. The results demonstrated an increase in cell proliferation and a decrease in cell apoptosis after irradiation. The intensity and activity of ALP staining were also significantly increased after irradiation. The level of mineralization was clearly enhanced in the irradiated groups compared to non-irradiated controls. qRT-PCR showed significant increases in the expression of mRNA of osteocalcin (OCN) and osteoprotegerin (OPG) in the irradiated groups, with no significant differences between the two wavelengths used [47].

Rosenberg et al. investigated cell proliferation, markers of cell maturation and metabolic activity after laser exposure. Cultures of human osteoblast-like cells were exposed four times, at 24 h intervals, for 2 min, to a radiation of 2.4–2.5 mW cm^2^. Cell proliferation was estimated by microscopic cell counting and cell death by lactate dehydrogenase activity in the culture medium (measured by a colorimetric method). Early markers of osteoblast maturation and metabolic activity, i.e., cellular alkaline phosphatase activity and osteocalcin content, were measured using a colorimetric method (ELISA). 40 Hz irradiation caused the greatest increase in cell number (*p* < 0.01). The content of osteocalcin in the cells decreased after 40 Hz and 10 Hz irradiation (*p* < 0.05). Irradiation in the blue range of 40 Hz (diffuse transmission 420–580 nm, maximum power 0.5 mW cm^2^) caused a decrease in cellular alkaline phosphatase activity (*p* < 0.001) and an increase in the average content of osteocalcin (*p* < 0.05). The 40 Hz irradiation range (diffuse transmission 560–650 nm, peak power 0.4 mW cm^2^) caused an increase in cell number and cell death. In conclusion, pulsed white light (40 Hz) irradiation has photo modulatory effects, its green spectrum affects cell proliferation and death, and its blue spectrum affects cell maturation and metabolism. The results indicate a low intensity threshold of photo biomodulation of osteoblast-like cells in vitro [48].

Cardoso et al. evaluated the photo-bio-stimulatory effects of laser (PBM) on mouse skull-derived osteoblasts (rGO) cultured on regular or osteogenic media. Cell cultures were exposed to different laser radiations: red laser (RL3-5 J/cm^2^, 3 s and RL5-8.3 J/cm^2^, 5 s, 1.66 W/cm^2^); infrared laser (IrL3-5 J/cm^2^, 3 s and IrL5-8.3 J/cm^2^, 5 s); LED (LED3-3 s and LED5-5 s, 0.02 J/cm^2^, 0.885 W/cm^2^). PBM with red laser and LED induced mineralization by itself without osteogenic medium, which was not observed for infrared laser (*p* < 0.05). The effects were found on osteogenic medium and PBM by infrared, red laser, and LED (5 s). Red laser and LED increased the proliferative, migratory, and secretory phases in rGO cells in a dose-dependent manner. Red laser and LED PBM promote osteogenic induction by itself. PBM with infrared laser and osteogenic medium potentiates mineralization [49].

Since there is still controversy on the utility of laser application on the periodontal and bone tissues of the oral cavity, our study sought to assess whether the use of a 980 nm laser could induce positive results on osteoblasts harvested from patients of different age groups and from different donor sites. Although this is not the only research performed on this topic, the fact that we obtained the bone material from adult patients who sought periodontal, implantary or orthodontic treatment represented the novelty of our study. Primary cells provide a more complex response and faster feedback to environmental stimuli in vitro. Therefore, the fact that we used human bone cells and not commercial cells gives a greater importance to our results.

The limitations of our study consist of the small study group; however, this was due to the strict inclusion and exclusion criteria. Another limitation was the application of only one wavelength of 980 nm on the cells.

## 5. Conclusions

The bone explant can be used to obtain mesenchymal stem cells, with the ability to proliferate and differentiate into osteoblasts, in a culture medium enriched with specific elements.

When analyzed with a confocal microscope, the fluorochrome for alkaline phosphatase appears in a much higher quantity than that for osteocalcin, a sign of the presence of early osteoblasts in the culture medium.

The quantification of cell proliferation identified a greater number of cells in the cell cultures from the laser group “+” compared to the laser group “-”, for all patients, with statistically significant differences (*p* = 0.0072). Cell proliferation was different from one patient to another, even if the working protocol was identical, being influenced by the age of the patient.

Laser application applied to osteoblast cultures contributes to increasing the proliferation rate and intensifying their cellular activity.

## Figures and Tables

**Table 1:** Parameters used during acquisitions with confocal equipment.

Fluorochromes	λ Excitation	Biological Sample	Biological Sample	LASER Power	Detector Type	Emission Band
Alexa Fluor 488	488 nm	Intracellular alkaline phosphatase	osteoblasts	7 mW	PMT	503–580 nm
Alexa Fluor 594	561 nm	Intracellular osteocalcin	osteoblasts	35.6 mW	HyD Intern	590–790 nm
DAPI	405 nm	Nuclear DNA	osteoblasts	24.5 mW	PMT	415–475 nm

**Table 2:** Parameters used during microscopic acquisitions with confocal equipment for viability assessment.

Fluorochromes	λ Excitation	Biological Sample	Biological Sample	LASER Power	Detector Type	Emission Band
Ethidium III Homodimer (EthD-III)	561 nm	Dead cells’ DNA	osteoblasts	20 mW	PMT	575–780 nm
Calcein AM	496 nm	Esterases from living cells	osteoblasts	8 mW	HyD Intern	505–611 nm

**Table 3:** Characteristics of the study group.

Patient	Age	Gender	Harvest Site
1	28	female	Upper second premolar (2.5)
2	22	male	Upper second molar (1.7)
3	20	female	Upper first premolar (1.4)
4	38	female	Upper third molar (2.8)
5	21	male	Lower second molar (4.7)
6	41	female	Lower third molar (3.8)
7	45	male	Lower second premolar (3.5)
8	49	female	Upper second molar (1.7)

**Table 4:** Characteristics of the osteoblast cultures.

Slice	Count (n)	Total Area (Pixels)	Average Size (Pixels)	Area (%)
Patient 1 laser +	2309	553,123.852	239.551	10.556
Patient 1 laser -	1315	1,401,762.407	1065.979	26.777
Patient 2 laser +	3701	645,989.447	174.545	12.292
Patient 2 laser -	2508	638,447.68	254.564	12.166
Patient 3 laser +	4617	537,615.553	116.443	41.039
Patient 3 laser -	2860	480,853.707	168.131	36.527
Patient 4 laser +	1537	239,764.02	155.995	18.231
Patient 4 laser -	482	133,835.969	277.668	10.216
Patient 5 laser +	4583	1,076,814.891	234.959	20.55
Patient 5 laser -	2478	795,648.215	321.085	15.206
Patient 6 laser +	1322	620,348.193	469.25	11.85
Patient 6 laser -	1156	432,137.329	373.821	8.259
Patient 7 laser +	529	1,772,710.067	335.105	16.906
Patient 7 laser -	220	489,080.086	222.309	3.722
Patient 8 laser +	241	739,000.588	306.641	5.541
Patient 8 laser -	93	2,791,200.064	300.129	0.665

## Data Availability

The data presented in this study are available on request from the corresponding authors. The data are not publicly available due to personal protection.
